# Calcium: The Missing Link in Auxin Action

**DOI:** 10.3390/plants2040650

**Published:** 2013-10-21

**Authors:** Steffen Vanneste, Jiří Friml

**Affiliations:** 1 Plant Systems Biology, VIB, and Plant Biotechnology and Bio-informatics, Ghent University, Ghent 9052, Belgium; 2 Institute of Science and Technology Austria (IST Austria), Klosterneuburg 3400, Austria

**Keywords:** auxin, calcium, signal transduction, auxin transport

## Abstract

Due to their sessile lifestyles, plants need to deal with the limitations and stresses imposed by the changing environment. Plants cope with these by a remarkable developmental flexibility, which is embedded in their strategy to survive. Plants can adjust their size, shape and number of organs, bend according to gravity and light, and regenerate tissues that were damaged, utilizing a coordinating, intercellular signal, the plant hormone, auxin. Another versatile signal is the cation, Ca^2+^, which is a crucial second messenger for many rapid cellular processes during responses to a wide range of endogenous and environmental signals, such as hormones, light, drought stress and others. Auxin is a good candidate for one of these Ca^2+^-activating signals. However, the role of auxin-induced Ca^2+^ signaling is poorly understood. Here, we will provide an overview of possible developmental and physiological roles, as well as mechanisms underlying the interconnection of Ca^2+^ and auxin signaling.

## 1. Auxin and Ca^2+^: Essential Elements of Plant Development

How do you survive when you are sessile? The answer is that you need to be extremely flexible and even change your body plan according to actual limitations imposed by the environment. Herein lays the key to the success of plants. They can develop highly complex and elaborate body plans under optimal conditions, while less favorable environments results in much smaller plants with reduced body plan complexity. The ability to adjust the number and size of organs, regenerate damaged or destroyed organs and to (re)orient growth according to light and gravity are a few examples of the mechanisms that illustrate plants’ flexible, adaptive growth. 

At the very core of this developmental flexibility is the plant hormone auxin [1,2,3]. Several decades of intensive research have revealed that this plant hormone is involved in nearly every aspect of plant development, ranging from embryogenesis and multiple steps of organogenetic processes in the root (lateral root initiation, morphogenesis and outgrowth, stem cell niche maintenance) [4,5] and shoot (phyllotaxis [6], leaf initiation [7], leaf morphogenesis [8], vascular patterning [9], ovule patterning [10]), but is also involved in apical hook formation [11,12], gravitropism [13], hydrotropism [14], phototropism [15,16,17], shade avoidance [18], root hair formation [19,20], stomatal opening [21], pollen development [22], senescence [23,24], fruit development [25,26], leaf abscission [27], response to pathogens [28,29] and abiotic stress [30]. At the cellular level, this is reflected in control over cell differentiation [31], cell cycle regulation [32,33,34], cellular morphogenesis (e.g., leaf pavement cells) [35] and membrane trafficking [36,37,38,39]. Because of auxin’s pleiotropic morphogenetic capacities, plants have elaborate mechanisms to prevent unwanted/unnecessary auxin activity. Together, regulation of biosynthesis, conjugation, subcellular compartmentalization, degradation and active transport act to control the cellular auxin levels [40,41]. Additional fine-tuning of auxin’s action is achieved by modulation of the signal transduction pathway(s) [42,43]. Via one or more of these control mechanisms, a myriad of endogenous (developmental and hormonal) and exogenous signals (stresses, nutrient availability, *etc*.) impact on auxin’s action, thereby optimizing the plant’s growth and development [44]. 

The divalent cation, Ca^2+^, is one of the most universal second messengers; a signal that relays a primary signal, such as derived from an activated receptor, from the surface of the cell to intracellular targets. It can be found in ancient bacteria and throughout eukaryotic lineages, where it is essential to support life [45,46]. The importance and pleiotropism of Ca^2+^ in cellular signaling processes is roughly summarized in the famous quote from Otto Loewi in 1959: “Ja Kalzium, das ist alles!” (Yes, calcium is everything!). Furthermore, in plants, Ca^2+^ is a fundamentally important second messenger, as demonstrated by its involvement in a multitude of essential cellular process, ranging from cell division, cell growth/shrinkage, secretion, transcriptional regulation, cellular polarity, *etc*., by which it impacts on stomatal aperture regulation, responses to light, responses to biotic and abiotic stresses, immunity a and responses to multiple plant hormones, including response to auxin [47,48,49]. Because Ca^2+^ signals can be regulated by so many different cues and have such a broad impact on cellular processes, it is not unlikely that Ca^2+^ acts to integrate multiple cues in a single output. Here, we will focus on the effects of auxin on Ca^2+^ and *vice versa*. 

## 2. The Source of Auxin-Induced Ca^2+^ Signals

Via Ca^2+^ sensitive dyes and, recently, also genetically encoded Ca^2+^ sensors, strong correlations could be observed between Ca^2+^ and auxin signaling. This is most apparent after exogenous application of synthetic and natural auxins, which induce a rapid, transient increase in cytosolic Ca^2+^ concentration in wheat leaf protoplasts [50,51], maize coleoptile and root cells [52,53,54], parsley hypocotyl segments [53], intact *Arabidopsis* roots [55] and closed orchid guard cells [21]. In nearly all cases, the increase of cytosolic Ca^2+^ concentration was observed to occur within minutes after auxin application, demonstrating that auxin is a potent inducer of Ca^2+^ signals. One of the important questions that remain to be answered is how these auxin-induced Ca^2+^ signals are generated. Generally, rapid and local Ca^2+^ signals generated in the cytosol depend on a Ca^2+^ current that is driven by a steep concentration gradient between the cytosol (nanomolar range) and its adjacent organelles or extracellular space (millimolar range). This allows the bringing about of a rapid and strong Ca^2+^ rise in the cytosol required for immediate activation of an appropriate response by the simple opening of a few Ca^2+^ channels [45,46,56].

In plants, the apoplast, the vacuole, the endoplasmic reticulum and all smaller organelles could serve as sources for auxin-induced Ca^2+^. The apoplast is an important source for auxin-induced Ca^2+^ signals. These could be prevented by application of membrane impermeable Ca^2+^ chelators and Ca^2+^ channel inhibitors [55,57], and auxin-induced Ca^2+^ changes in leaf wheat protoplasts and root hairs depend strongly on the Ca^2+^ concentration in the extracellular medium [50,51,58]. Moreover, the direct involvement of plasma membrane-localized channels could be measured in plasma membrane-derived vesicles [59,60]. In these experiments, vesicles that consist of 70%–80% of plasma membrane were prepared from maize coleoptiles. Auxin-induced changes in membrane potential were then measured in the context of a different concentration of intraluminal and extraluminal K^+^ and Ca^2+^. From these measurements, it was inferred that auxin activates cation channels in the plasma membrane that facilitate Ca^2+^ influx and K^+^ efflux. Consistently, both auxin-induced currents could be inhibited by nifedipine and verapamil [59,60], two Ca^2+^ channel blockers that were reported to inhibit outward rectifying K^+^ channels [61]. Together, these findings strongly support the involvement of plasma membrane-localized Ca^2+^ channels to generate auxin-induced Ca^2+^ signals. Importantly, a large portion of wheat leaf protoplasts showed a LiCl-sensitive, biphasic Ca^2+^ signal after auxin treatment, suggesting the involvement of intracellular Ca^2+^ stores [51]. However, it remains to be further explored how intracellular Ca^2+^ stores contribute to auxin-induced Ca^2+^ signals. Unfortunately, most of the used dyes and genetically encoded Ca^2+^ sensors did not yield the needed resolution to detect intracellular sources for auxin-induced Ca^2+^. Recently, the genetically encoded Ca^2+^ sensor, aequorin, was targeted to the Golgi apparatus and revealed that application of the synthetic auxin analogue, 2,4-D (2,4-dichlorophenoxyacetic acid), caused a steady/slow decrease of its Ca^2+^ content, suggestive of a passive Ca^2+^ loss [62]. To be able to further explore the contributions of the different organelles to auxin-induced Ca^2+^ fluxes, one would require using a broad range of subcellularly targeted Ca^2+^ sensors that were recently developed [62,63,64,65,66]. 

## 3. Auxin Receptors for Auxin-Induced Ca^2+^ Signals

**Figure 1:**
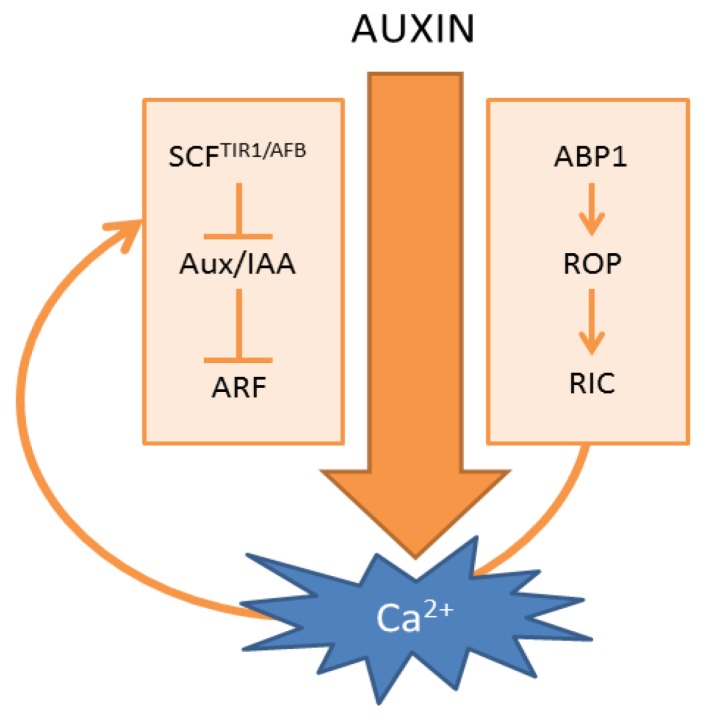
Scheme of auxin-induced Ca^2+^ signals. (**Left**) Canonical SCF^TIR1/AFB^-mediated auxin signaling; (**Right**) ABP1-mediated auxin signaling. The curved arrow represents a hypothetical model in which Ca^2+^ acts as a connecting signal between ABP1 and SCF^TIR1/AFB^ signaling cascades.

Several decades of extensive auxin research allowed the mapping of the main auxin signaling pathways. First, and best characterized, are the auxin-induced transcriptional changes that reflect auxin-driven developmental decisions [1]. This pathway is defined by the SCF^TIR1/AFB^ E3-ligase, Aux/indole-3-acetic acids (Aux/IAAs) and auxin response factors (ARFs) (Figure 1). The Aux/IAAs are repressors of auxin response factors, which recruit the TOPLESS (TPL) co-repressor to these transcription factors. Under high auxin conditions, the interaction between the Transport Inhibitor Response1/Auxin Signaling F-Box (TIR1/AFB) component of the E3-ligase and Aux/IAAs is stabilized, resulting in ubiquitination of the Aux/IAA and its subsequent proteasomal degradation. The rapid proteolysis of Aux/IAAs results in the derepression of ARFs and associated transcriptional changes [43]. However, the speed by which auxin can elicit Ca^2+^ signals precludes the involvement of transcriptional changes, suggesting that it acts independently from the canonical SCF^TIR1/AFB^-based auxin signaling cascade. This notion can also be deduced indirectly from the available literature on auxin-induced pH changes. Within minutes, auxin induces a rapid acidification of the cytosol [21,54,67,68] and an alkalinization of the apoplast in the root cells of *Arabidopsis* [55]. Interestingly, the auxin-induced apoplast alkalinization of *Arabidopsis* root cells occurs equally fast in wild-type as in *tir1* and *tir1afb2ab3* mutants [55], suggesting that this process is SCF^TIR1/AFB^ independent. On the other hand, both auxin-induced Ca^2+^ and auxin-induced apoplast alkalinization were abolished in the presence of the general Ca^2+^ channel inhibitor, La^3+^ [55]. This together suggests a Ca^2+^-dependence of the apoplast alkalinization response, which is SCF^TIR1/AFB^ independent.

An alternative auxin signaling pathway based on the stabilized interaction between F-box protein SKP2A and cell cycle transcription factor DPB was recently proposed to explain the effects of auxin on cell cycle progression [69]. However, as this would also act in the nucleus to drive transcriptional changes, it also seems unlikely that this pathway could account for auxin-induced Ca^2+^ changes. 

A second main auxin receptor is defined by Auxin Binding Protein 1 (ABP1) [3,70,71,72]. This protein resides mainly in the endoplasmic reticulum (ER), where it is probably unable to bind auxin, due to the high pH. A small fraction of ABP1 seems to escape from the ER via the secretory pathway, to the more acidic apoplast, which is more favorable for auxin binding [73]. In the apoplast, it remains closely associated with the plasma membrane, where it could modulate auxin responses that do not require transcriptional changes, such as membrane hyper/depolarization [74,75], regulation of auxin-induced currents of K^+^ and Cl^−^ across the plasma membrane [76,77] and regulation of clathrin-mediated endocytosis [39,78,79,80]. Recently, it was found that ABP1 is required for the auxin-mediated activation of Rho of Plant (ROP) GTPases, which exert their effects through interaction with ROP interactive CRIB (Cdc42/Rac Interactive Binding) motif-containing proteins (RIC) [78] (Figure 1). As ABP1 controls fast auxin responses at the plasma membrane, one might expect that ABP1 also acts upstream of auxin-induced Ca^2+^ signaling. However, available data are indirect and inconclusive as exemplified in auxin-induced stomatal opening. Firstly, it is well established that auxin induces stomatal opening [81], which has been correlated with the induction of Ca^2+^ signals and cytosolic acidification in guard cells [21]. Secondly, lowering Ca^2+^ via ethylene glycol tetraacetic acid (EGTA) prevented auxin-induced stomatal opening [57] indicating that apoplastic Ca^2+^ is required for auxin-induced stomatal opening. Thirdly, activation of apoplastic ABP1 via exogenous application of a specific antibody induced stomatal opening, while exogenous application of polyclonal antibodies could interfere with auxin-induced stomatal opening [82]. Together, these findings are consistent with a model in which extracellular ABP1 acts upstream of Ca^2+^ during auxin-induced stomatal opening. However, in mutants defective in AUX1-mediated IAA uptake, IAA could no longer counteract abscisic acid (ABA)-induced stomatal closure [83], arguing against the involvement of an extracellular auxin receptor. Moreover, the Ca^2+^-dependent portion of auxin-induced protoplast swelling was suggested to be independent of apoplastic ABP1 [84]. 

The ABP1 and SCF^TIR1/AFB^ signaling cascades are interconnected: in conditional knockdown lines for ABP1, SCF^TIR1/AFB^-regulated transcripts were less auxin-sensitive [34], and TIR1/AFB-dependent Aux/IAA degradation was enhanced [85]. While the underlying mechanism is currently unknown, one could speculate that Ca^2+^ acts as a coordinating signal between both signaling cascades (Figure 1). Indeed, the early auxin-responsive gene, *TaIAA1*, could be controlled by Ca^2+^ application, which was prevented by Ca^2+^ chelation [86], suggesting that the SCF^TIR1/AFB^ signaling cascade can be controlled by Ca^2+^ signals, which are potentially generated downstream of ABP1.

## 4. Calcium Modulates pH and Growth

After the discovery of the molecular nature of auxin, it was readily recognized that auxin can promote or inhibit growth depending on its concentration [87]. At low concentrations, auxin stimulates growth, while high auxin concentrations repress growth. 

Auxin-induced growth can be largely explained by a rapid elongation of cells and is correlated with acidification of the apoplast [88], activation of cell wall modifying enzymes [89,90] and K^+^ uptake [91,92,93]. These elements have been rationalized in the “Acid Growth Theory” [94,95,96,97,98,99], which suggests that apoplast acidification is the major regulator of auxin-induced elongation by activating cell wall loosening enzymes and by providing the electrochemical gradient that drives K^+^ uptake, which is necessary for water uptake and cell expansion. This theory provides a nice overview of events that correlate with auxin-induced growth, but remains controversial, due to a lack of strong biochemical and molecular support.

**Figure 2:**
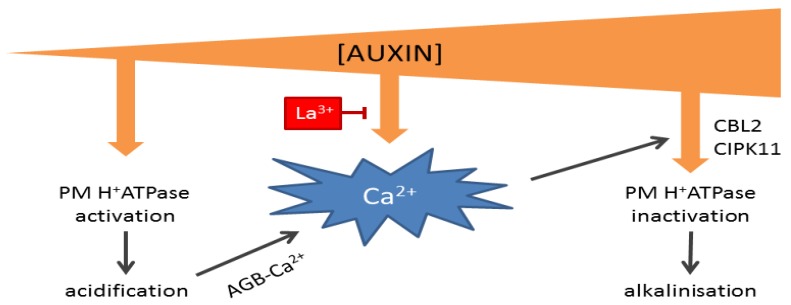
Hypothetical model of auxin concentration-dependent control over apoplastic pH. At low concentrations, auxin activates plasma membrane (PM) H^+^ ATPases, thereby lowering apoplastic pH and increasing apoplastic Ca^2+^ concentrations via arabinogalactan glycoproteins (AGBs). At high auxin concentrations, auxin induces a Ca^2+^ signal that inactivates H^+^ ATPases. The auxin-induced Ca^2+^ signal can be inhibited by La^3+^.

Central to the activation of plasma membrane-localized H^+^ ATPases (AHAs) is the phosphorylation-dependent interaction with a 14-3-3 protein. This interaction, and, thus, proton extrusion, depends mainly on phosphorylation of the penultimate Thr in its *C*-terminus [100,101], which is also targeted during auxin-induced elongation and occurs with a lag of ~10 min after auxin application [88]. On the other hand, the interaction between AHA2 and the activating 14-3-3 protein can be inhibited by phosphorylation of a Ser-931 in its *C*-terminus by the protein kinase PKS5/CIPK11, which acts in concert with the Ca^2+^ binding protein, ScaBP1/CBL2 [102], and could explain the Ca^2+^-dependent root growth inhibition in high auxin levels [103,104]. Importantly, this Ca^2+^-dependent inhibition could be part of a feedback mechanism that keeps apoplast acidification in check, as arabinogalactan glycoproteins (AGBs) are proposed to act as pH-sensitive Ca^2+^ sources in the periplasm [105]: Acidification of the apoplast would thus increase the unbound Ca^2+^ concentration that can contribute to cytosolic Ca^2+^ signals that inhibit AHA activity (Figure 2).

The existence of two antagonistic pathways for regulating AHA activity with two different auxin sensitivities would provide an easy explanation for the observed concentration-dependent dualism of auxin as a regulator of elongation growth [87] (Figure 2). At suboptimal concentrations, auxin would mainly stimulate AHA activity to drive elongation, until a threshold concentration at which auxin triggers Ca^2+^-dependent inhibition of AHAs. The auxin receptor for these responses might be ABP1, as auxin-induced elongation and H^+^ ATPase phosphorylations are independent of the canonical SCF^TIR1/AFB^ signaling pathway [88,106], and antigenic inhibition of ABP1 can prevent auxin-induced H^+^ ATPase activity [72,75,107].

Besides a possible modulation of H^+^ ATPase activity, Ca^2+^ is well known to control K^+^ uptake via direct regulation of K^+^ channels in guard cells and in roots [108,109,110,111,112,113]. In these examples, Ca^2+^ sensing proteins of the calcineurin B-like (CBL) family, together with CBL interacting kinases (CIPK) or Ca^2+^-dependent kinases (CPKs), mediate Ca^2+^-dependent phosphorylation of K^+^ channels to fine-tune their activities. Furthermore, during auxin’s effect on osmoregulation, Ca^2+^ could be an important signal, as high extracellular Ca^2+^ impairs acid-induced growth, which is associated with reduced inward K^+^ currents [93]. Moreover, K^+^ transporters, TRH1/AtKT3/AtKUP4 [114,115] and ZIFL1 [116], and CIPK6 [117] (which can phosphorylate the K^+^ transporter AKT2 [118]) have been connected to the regulation of auxin transport. Therefore, the effect of Ca^2+^ on K^+^ channels could be related to regulating osmotic pressure for both cell elongation and for auxin transport. 

## 5. Ca^2+^ Controls the Rate of Auxin Transport

As early as the nineteenth century, Charles Darwin already recognized the existence of a mobile signal that moves between the site of light perception and the site of elongation growth during phototropic bending [119]. Ever since the discovery of auxin, auxin transport has been recognized as a crucial aspect of auxin-regulated growth [2]. Two types of auxin transport can be distinguished. The first is passive, long-distance auxin transport via the vascular tissues for source-to-sink auxin transport [120]. The second is slower, directional (polar), cell-to-cell transport for auxin-regulated plant development. The latter is one of the main mechanisms by which instructive auxin gradients in tissues are formed to regulate plant development. Interestingly, polar auxin transport was found to be highly dependent on Ca^2+^ availability [104,121,122,123,124,125]. Together, these findings highlight the importance of Ca^2+^ in auxin transport. 

**Figure 3:**
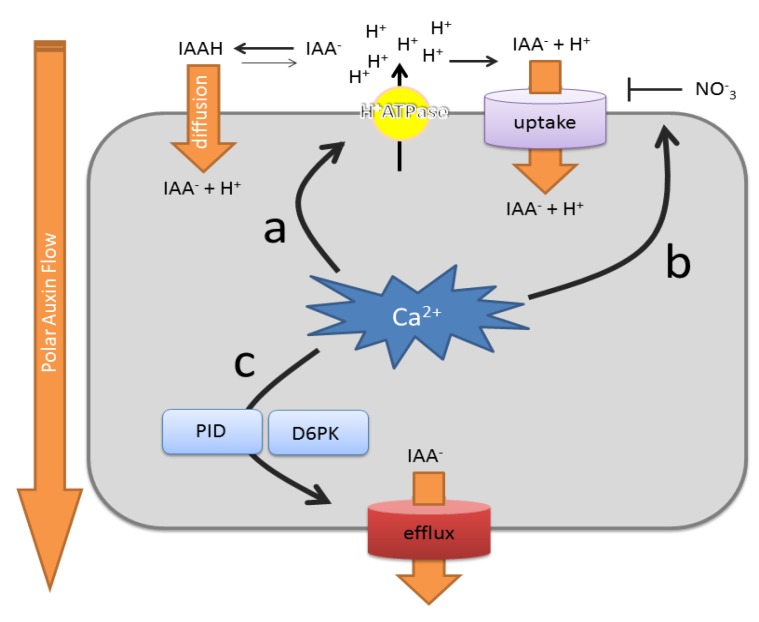
Summary of the effects of Ca^2+^ on polar auxin transport rates. (**a**) Cellular Ca^2+^ signaling impacts on auxin uptake mechanisms via effects on the abundance and activity of the plasma membrane H^+^ ATPase. The amount of protons in the apoplast determine the auxin uptake rate via diffusion of protonated indole-3-acetic acid (IAAH), as well as H^+^/IAA^-^ (indole-3-acetic acid) symport; (**b**) Ca^2+^ can change the affinity of NRT1.1 for nitrate and auxin uptake; (**c**) Ca^2+^ controls the activity of the auxin efflux machinery by modulating the kinase activity of PINOID (PID) and, possibly, also D6PKs. PID also impacts on PIN-formed (PIN) polarity (not depicted). Uptake refers to active uptake mechanisms. Efflux refers to active auxin efflux mechanisms.

The minimal mechanistic constituents of polar auxin transport have been delineated in the chemiosmotic polar diffusion hypothesis [126,127,128,129] (Figure 3). This model states that the natural auxin, indole-3-acetic acid (IAA), in the acidic environment of the apoplast exist (in part) in its protonated form, which renders it more lipophilic and, thus, allows it to diffuse through the plasma membrane. Once inside the neutral cytosol, it loses its lipophilicity by deprotonation and is trapped inside the cell. The rate of auxin efflux from the cell is, therefore, dependent on the activity of auxin efflux proteins. By extension, this model predicts that asymmetric localization of such auxin efflux transporters could explain polar auxin transport across tissues. 

Since the formulation of this model, most of the underlying molecular machinery has been identified and characterized in detail. Besides passive auxin entry, auxin was found to be actively taken up into the cell (Figure 3). This is mainly mediated by auxin influx transporters of the AUX1/LAX family [130,131], at least one member of the ABCB family [132] and the nitrate sensor/transporter, NTR1.1 [133]. The AUX1/LAX proteins are amino acid permease-like proteins that act as high affinity H^+^/IAA^−^ symporters [134]. They are involved in efficient auxin uptake into the cell, which is needed for gravitropism [131,135], lateral root emergence [136] and phyllotaxis [137]. Interestingly, vacuolar Ca^2+^ was found to have a profound, but indirect, impact on AUX1-mediated H^+^/IAA^−^ symport, via effects on plasma membrane H^+^ ATPase activity [83] (Figure 3a). Mutants defective in the vacuolar Ca^2+^/H^+^ exchangers, CAX1 and CAX3, were insensitive to IAA’s inhibitory effect on ABA-induced stomatal closure. This defect in IAA sensitivity could be fully rescued by the lowering of apoplastic pH in the mutants. This illustrates how vacuolar Ca^2+^ homeostasis processes can impact on auxin uptake. Similarly, *cax1* mutants show reduced lateral root densities and show IAA-resistant root growth [138], reminiscent of *aux1* mutant phenotypes [120,139], suggesting that a similar mechanism as described for stomata can explain the observed root phenotypes.

While AUX1/LAX transporters mediate the bulk of the auxin influx into the cell, ABCB4, its close homologue, ABCB21, and NRT1.1 represent conditional auxin uptake mechanisms. On the one hand, ABCB4 and ABCB21 mediate auxin uptake when auxin levels are low, but catalyze auxin efflux at higher concentrations [132,140]. However, no effects of Ca^2+^ on their auxin transport activity were thus far reported. On the other hand, the NRT1.1/CHL is a dual-affinity nitrate transporter, which also serves as a nitrate sensor [141]. This protein was recently reported to also facilitate auxin uptake [133]. Importantly, NRT1.1-dependent auxin uptake could be inhibited by nitrate, highlighting a direct cross-talk between nutrient sensing and auxin transport. Moreover, NRT1.1’s affinity for nitrate is controlled by CIPK23-mediated phosphorylation [141], suggesting that Ca^2+^ signals control auxin uptake via modulating NRT1.1’s affinity for nitrate (Figure 3b). 

The other rate-limiting aspect of the polar auxin transport is auxin efflux (Figure 3c). This process is mainly mediated by auxin transporters of the PIN-formed (PIN) family and a subgroup of the ABCB transporter family [142,143]. They can transport auxin independently, but also in concert with each other [144,145,146,147]. Among the ABCB transporters, ABCB1, ABCB4, ABCB19 and ABCB21 are best characterized for their auxin-transport capacities [140,148,149]. Their auxin efflux activities are stimulated by interaction with the immunophilin-like TWISTED DWARF1 (TWD1) [150,151], and this interaction is inhibited by synthetic and natural auxin transport inhibitors [140,146]. Besides the interaction with TWD1, ABCB1 auxin efflux activity can be impaired or stimulated by phosphorylation via the AGC kinase, PINOID (PID) [146]. In the presence of TWD1, PID-mediated phosphorylation inhibits auxin efflux, whereas it acts in a stimulatory manner in the absence of TWD1. On the other hand, PIN-mediated auxin transport rates can be regulated by D6PK-mediated phosphorylation, another subclade of AGC kinases [152]. Interestingly, PID kinase activity can be enhanced or repressed by interaction with the Ca^2+^-binding proteins, PID-BINDING PROTEIN 1 (PBP1) and TOUCH 3 (TCH3), respectively [153]. This suggests that Ca^2+^ can have a positive, as well as a negative, impact on PID activity and, thus, on ABCB-mediated auxin transport. Conversely, as D6PKs are alsoAGC-type kinases, it is tempting to speculate that these kinases could also be regulated by Ca^2+^-binding proteins to regulate PIN-mediated auxin transport activity. 

These examples demonstrate how Ca^2+^ could impact on auxin transport rates via direct effects on the auxin transport machinery. However, there are probably even more mechanisms by which Ca^2+^ can impact on polar auxin transport. One example of this is the recent finding that overexpression of SAUR19, a member of an early auxin responsive protein family that binds with high affinity to calmodulin [154], promotes cell expansion and polar auxin transport by an unknown mechanism [155].

## 6. Ca^2+^ in the Balance of Exocytosis and Endocytosis

While the above examples demonstrate how Ca^2+^ impacts on the speed of auxin transport, it was already proposed in 1984 that reduced polar auxin transport under low Ca^2+^ conditions was the result of both a lower velocity and a lower capacity for auxin transport [156]. This implies that Ca^2+^ controls not only the activity, but also the relative abundance of auxin transporters at the plasma membrane. 

Newly biosynthesized PINs are trafficked from the endoplasmic reticulum, via the Golgi apparatus and *trans*-Golgi Network (TGN) to the plasma membrane. Via clathrin-mediated endocytosis, PIN proteins are removed from the plasma membrane to early endosomes/TGN from which they can be targeted to the vacuole for degradation or recycled for exocytosis at the PM [157]. Thus, the predicted impact of Ca^2+^ on PIN abundance at the plasma membrane is determined by the balance between exocytosis and endocytosis.

One of the most famous effects of Ca^2+^ on membrane trafficking in animals is the activation of exocytosis during neurotransmission [158] and hormone secretion [159]. In plants, Ca^2+^ is also intimately connected to regulated exocytosis, as exemplified in gibberellic acid-induced alpha-amylase secretion [160,161], peroxidase secretion [162,163] and polar growth [164,165,166,167]. The stimulatory effect of Ca^2+^ on exocytosis could also be directly observed by Ca^2+^-induced increases of membrane capacitance in protoplasts of barley aleurone cells [168,169], maize coleoptiles [170,171], maize root caps [172] and tobacco calli [173]. Additionally, Ca^2+^ might also increase secretion by stimulating *de novo* synthesis of secretory cargoes [173,174]. That Ca^2+^ could be involved in PIN secretion would be a plausible assumption; however, without supporting experimental evidence, it remains equally plausible that PINs are constitutively secreted, in a Ca^2+^-independent manner. 

**Figure 4:**
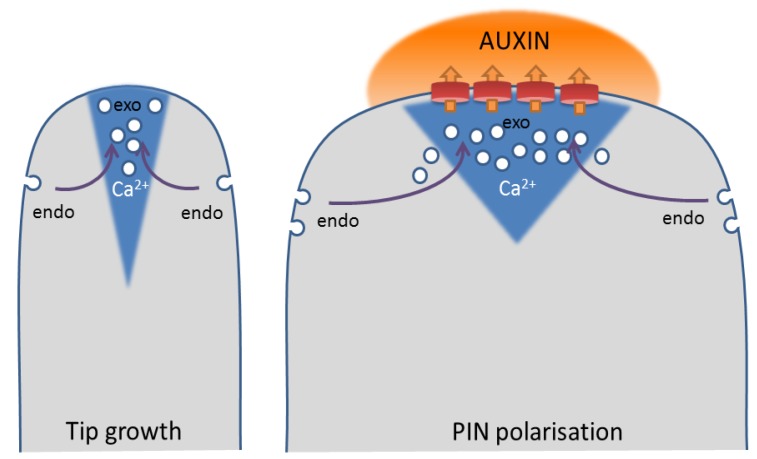
Scheme of membrane trafficking in tip growth and PIN polarization. (**Left**) Scheme of a tip-growing cell, with high secretion rates centered on a tip-focused Ca^2+^ gradient and high endocytosis rates at the shank of the cell; (**Right**) A cell with polarized PINs at its apical cell side shows high rates of PIN endocytosis at its flanks and polar recycling to the center of its apical domain. Polarized auxin efflux locally causes an increase in auxin concentration, which could elicit Ca^2+^ signals in the underlying cytoplasm. (endo = clathrin-mediated endocytosis; exo = exocytosis)

Indications for the involvement of Ca^2+^ as a coordinator of PIN trafficking derive from the spatial separation of clathrin-mediated endocytosis and exocytosis of polarized PINs [175], which is reminiscent of a polarized tip growth in pollen tubes and root hairs (Figure 4). During tip growth, a tip-focused Ca^2+^ gradient coordinates secretion, endocytosis and actin dynamics [167,176,177]. Secretory vesicles are polarly delivered to the growing tip via filamentous actin (F-actin), where they cannot fuse to the plasma membrane until the cortical F-actin is depolymerized to allow vesicle docking and fusion [178]. The tip-focused, oscillating Ca^2+^ induces F-actin depolymerization via activation of ABP29 in lily pollen [179], thereby stimulating exocytosis. During tip growth, too much membrane material is delivered compared to what is necessary for the fast, expansive growth [180]. Therefore, a considerable amount of materials, including regulators, are recycled by endocytosis. In pollen, two types of endocytosis could be distinguished: in the shank and subapical region, clathrin-mediated, actin-dependent endocytosis occurs, whereas in the apex, bulk endocytosis is actin-independent [181,182,183,184,185]. Thus, the sites of secretion and that of clathrin-mediated endocytosis coincide with high and low Ca^2+^ concentrations, respectively, suggesting that Ca^2+^ not only stimulates exocytosis, but simultaneously inhibits clathrin-mediated endocytosis. 

Therefore, auxin-induced Ca^2+^ could simultaneously induce secretion and inhibit clathrin-mediated endocytosis of PINs, thereby increasing the local auxin transport capacity, resulting locally in higher auxin concentrations, which, in turn, activate Ca^2+^ signaling. Such a positive feedback mechanism is consistent with our current models of auxin-regulated PIN polarization [175,186]. Thus far, it has been established that auxin can enhance its own efflux by inhibition of clathrin-mediated endocytosis of PINs [38]. Upon perception of auxin, extracellular ABP1 activates Rho of plant (ROP) GTPases to inhibit clathrin-mediated endocytosis via regulation of the actin cytoskeleton [39,78,79,80]. Recently, Ca^2+^ was placed upstream of ROP activity in pavement cells, as Rho GDI1 activity depends on phosphorylation by Ca^2+^-dependent kinase, CPK3 [187]. However, ROP GTPases could also act upstream of Ca^2+^, as the pollen-specific ROP interactor, RIC3, controls the tip-focused Ca^2+^ gradient in pollen tubes [188].

Such interplays between Ca^2+^ and ROPs are potential mechanisms by which signals, such as auxin [37,38], cytokinins [189], strigolactones [190], gibberellins [191,192], salicylic acid [193], *etc*., could control the turn-over of PINs to change auxin transport.

## 7. Ca^2+^ As a Means to Change Auxin Flow Direction

The plasma membrane-localized PIN proteins show typical asymmetric distribution patterns [2,194] that dictate the auxin flow direction within a given cell [195]. These subcellular polarities are not static, as they can be dynamically rearranged in response to endogenous [7,9,25,196] and exogenous signals [15,36,197,198]. 

Via misexpression studies, it was found that PIN polarities are not only determined by the cell type, but also by cues embedded in the structure of the PIN itself [195]. These polarity-determining signals within the PIN structure can be explained by specific phosphorylations in their hydrophilic loop [199,200] controlled by PINOID [201] and its counteracting phosphatase (PP2A) [202]. Phosphorylation by PINOID impacts on the subcellular trafficking of PINs by modulating their differential recruitment to distinct trafficking routes. In the root, PID-mediated phosphorylation renders PINs insensitive to GNOM-dependent trafficking, resulting in an apical (shootward) polarization [203]. During photostimulation, perception of light represses PID activity, allowing PIN3 to be recruited into GNOM-dependent trafficking toward the inner-lateral side of the cell [16]. Similarly, PID and GNOM activity are involved in PIN3 repolarization during shoot gravitropism [197]. That PID activity can be regulated by interaction with different Ca^2+^ binding proteins [153] suggests that Ca^2+^ signals could control PIN polarization via effects on PID activity. Indeed, important Ca^2+^ signals roughly coincide with PIN polarity changes during phototropism and gravitropism [15,16,197,204,205,206]. Moreover, mutations or treatments that lead to elevated Ca^2+^ levels were associated with shifts in PIN polarity [207]. 

## 8. The Ca^2+^-Auxin Interplay during Gravitropism

Gravitropism is an excellent example in which the interplay between auxin and Ca^2+^ is particularly apparent. Gravistimulation induces transient Ca^2+^ signals in maize coleoptiles [208], whole *Arabidopsis* seedlings [209], *Arabidopsis* leaf petioles and hypocotyls [210] and *Arabidopsis* roots [55]. The auxin dependence of these gravitropism-associated Ca^2+^ changes was demonstrated by genetic [55] and pharmacological interference with auxin transport [209,210]. Via a highly sensitive, genetically encoded Ca^2+^ sensor, Yellow Cameleon 3.60, a wave of Ca^2+^ was visualized that spread across the lower side of the root within minutes after gravistimulus [55], correlating spatially and temporally with the reported dynamics of auxin redistribution [211]. Not only cytosolic Ca^2+^ showed dynamics that correlate with auxin transport, but also auxin transport-dependent directional movement of Ca^2+^ across the gravistimulated tissues could be detected [212,213]. The gravistimulus-induced Ca^2+^ signals are particularly relevant, as gravitropic bending is severely impaired upon chelation of apoplastic Ca^2+^, inhibition of calmodulin or Ca^2+^ channels [214,215,216,217,218,219]. This illustrates the importance for Ca^2+^ in gravitropic bending. However, the underlying molecular mechanism remains poorly understood and, in some cases, even controversial [13,220]. 

**Figure 5:**
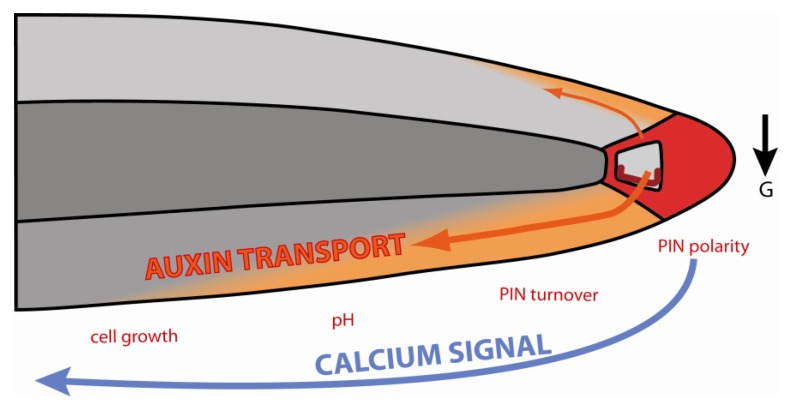
Interplay between auxin transport and calcium signaling during root gravitropism. Graviperception causes PIN repolarization in the columella, redirecting auxin flow to the lower side of the root. The new auxin flux is associated with local Ca^2+^ signals, altered PIN polarity and turnover, pH changes and inhibition of elongation. (The black arrow indicates the direction of gravistimulus).

A potential target of gravistimulus-induced Ca^2+^ signals is the plant’s ability to redirect auxin transport in response to the gravistimulus. Within minutes after graviperception, PIN3 and PIN7 root columella cells repolarize towards the direction of the gravitational pull [15,221], thereby enhancing auxin transport to the new lower side of the root (Figure 5). This additional auxin is efficiently taken up in root cap and epidermal cells via AUX1-mediated H^+^/IAA^-^ symport [134,135], to allow rapid efflux towards the root elongation zone via apically localized PIN2 and apical, PID-activated ABCB auxin transporters [146,222,223]. The increased auxin flux via PIN2 is capacitated by transient inhibition of endocytosis, which increases PIN2 abundance at the plasma membrane [37,38]. Simultaneously, the reduced auxin flows across the upper side of the root meristem and destabilizes PIN2 at the plasma membrane [36,37]. These complex effects on auxin transport differentially regulate auxin between the lower and upper side of the root elongation zone, differentially regulating elongation-driven growth and, thus, root bending. When *Arabidopsis* roots reach about the mid-point of bending, the asymmetry in auxin distribution is rapidly lost [211] and is associated with a neutralization of the imbalance of PIN2 abundance by auxin-induced PIN2 degradation [37]. 

Nearly every step of this summary of auxin-dependent root gravitropism has been highlighted in the paragraphs before as potentially regulated by Ca^2+^ and can be summarized in the following hypothetical model: Calcium signaling during graviperception activates PIN3 and PIN7 relocation in the columella to redirect auxin flow to the lower side of the gravistimulated root. Here, auxin induces Ca^2+^ signals that enhance auxin transport by impacting on trafficking, polarity and, even, the activity of the auxin efflux machinery (Figure 5). Moreover, the increase in auxin concentration interferes with elongation growth via inhibition of H^+^ ATPase activity, resulting in differential growth that is associated with root bending.

## 9. Conclusions and Future Perspectives

Following an era of physiological approaches, the auxin field shifted its attention towards elucidating the mechanisms of auxin-regulated transcription. This move has proven to be very successful with the genetic and molecular characterization of SCF^TIR1/AFB^-based auxin signal transduction. Numerous aspects of plant development can now be explained via this pathway and are fully justifying the efforts invested. Yet, it is clear that our understanding of auxin signaling will never be complete by only studying auxin-regulated transcription. Recent work on leaf pavement cell morphogenesis and feedback regulation of auxin transport highlighted the non-transcriptional effects of auxin as important aspects of general auxin physiology. Therefore, it will be of interest to revisit some of these physiological experiments in the context of more recent models of auxin action, armed with a new array of cell biological, genetic and molecular tools to gain more holistic insight into the mechanism of auxin-regulated plant growth and development. 
